# Anticipating Urgency: Predictive Factors for Early Intervention in Appendicular Perforation

**DOI:** 10.7759/cureus.74118

**Published:** 2024-11-20

**Authors:** Arun K Singh, Shivani B Paruthy, Vaibhav Kuraria, Inzamam Ul Hoda, Dhananjay Khera, Mohit Dhawaria, Hinduja Raju, Abhinav Kumar, Singamsetty S Madhuri, Yogesh Saini

**Affiliations:** 1 General Surgery, Vardhman Mahavir Medical College and Safdarjung Hospital, New Delhi, IND; 2 Medicine, Maulana Azad Medical College, New Delhi, IND

**Keywords:** abdominal sepsis, acute appendicitis diagnosis, acute perforated appendicitis, diagnosis of acute appendicitis, perforated appendix vermiform

## Abstract

Background: Appendicular perforation is a severe complication of acute appendicitis, leading to increased morbidity and complex post-surgical outcomes. Early identification of patients at risk of perforation is crucial to improve clinical management and reduce complications. This study aims to review and summarize the predictive value of clinical, biochemical, and radiological factors in determining the likelihood of appendicular perforation.

Methods: A comprehensive literature review was conducted to analyze key clinical, biochemical, and radiological markers associated with appendicular perforation. Clinical factors such as symptom duration, fever, and physical signs of peritonitis were examined. Biochemical markers, including white blood cell (WBC) count, hemoglobin, and serum creatinine, were evaluated for their diagnostic accuracy. Radiological imaging techniques, such as ultrasound (US), were assessed for their ability to detect perforation-related complications.

Results: This study identifies key predictors of appendicular perforation, including age, pulse rate, abdominal rigidity, and peri-appendiceal collection. Peri-appendiceal collection emerged as a strong predictor (OR=7.09). Logistic regression and machine learning models showed moderate predictive power (AUC 0.54-0.58), with demographic trends influencing clinical outcomes.

## Introduction

Acute appendicitis is among the most prevalent surgical emergencies, with an incidence ranging from 100 to 200 cases per 100,000 population annually. The incidence of appendicular perforation varies, but it has been reported to occur in 20%-30% of cases of acute appendicitis [1]. Perforation of the appendix is a serious complication that can lead to significant morbidity and mortality. Identifying the risk factors for appendiceal perforation is crucial for early diagnosis and appropriate management [2].

A range of pathological changes can occur in the appendix, leading to perforation. In the initial stages of appendicitis, there is inflammation and edema of the appendiceal wall, which can progress to necrosis and perforation if left untreated. The appendix may appear grossly enlarged, hyperemic, and edematous. With further progression, the appendiceal wall becomes thinner and may eventually rupture, leading to the spillage of appendiceal contents into the peritoneal cavity [3,4].

Accurate and early identification of patients at risk for appendicular perforation is crucial for optimizing clinical outcomes. However, the diagnosis of appendicitis and its progression to perforation can be complicated by several factors. Clinical presentation often varies widely, ranging from mild abdominal discomfort to severe pain, and can mimic other abdominal conditions, leading to diagnostic uncertainty [5]. Additionally, atypical presentations are more common in specific patient populations, such as the elderly, children, and pregnant women, further complicating the diagnostic process and delaying appropriate surgical intervention [6]. A study shows that abdominal pain in the appendicular lump is not always present and is limited to less than 50% of the cases [7]. As a result, there is a need to better understand the factors that predict early surgical intervention to mitigate the risks associated with perforation.

Several studies have investigated clinical and laboratory variables that may help distinguish perforated from non-perforated appendicitis in the pediatric population [2,8]. One retrospective analysis identified three independent predictors of perforation: duration of symptoms greater than one day, presence of fever in the emergency department, and elevated absolute neutrophil count on complete blood count [2]. Similarly, another study found that prolonged duration of symptoms, increasing temperature, and higher white blood cell (WBC) count were associated with a higher risk of appendiceal perforation [8].

Timely surgical intervention is critical in preventing complications of appendicitis. Progressive abdominal pain and abdominal rigidity, high-grade fever, tachycardia, and a raised total leukocyte count (TLC) after the first 24 hours may suggest progression to complications like perforation, which warrants urgent operative management. Conversely, in patients without evidence of perforation, a conservative approach with antibiotics may be appropriate in select cases.

In addition to the standard appendectomy, management of perforated appendicitis may require more extensive surgery in the form of laparotomy to address the complications of perforation, such as cecal blow-out and pyo-peritoneum/abscess formation. While small, well-contained perforations may be managed conservatively, severe cases often require invasive procedures such as abscess drainage, bowel resection, hemicolectomy, or diversion [9]. Prompt diagnosis and appropriate surgical intervention are critical to prevent the serious sequelae of appendicular perforation.

Furthermore, the identification of these predictive factors could facilitate the use of tailored treatment strategies, such as expedited diagnostic imaging or early surgical referral, particularly in high-risk patient groups. It could also potentially reduce healthcare costs associated with prolonged hospitalizations and complex postoperative care by preventing perforation before it occurs. Thus, this study aims to conduct a comprehensive analysis of the factors associated with early surgical intervention in cases of appendicular perforation. By synthesizing data from multiple sources, we aim to develop a clearer understanding of the variables that predict perforation, which could ultimately inform clinical guidelines and improve patient care.

The use of machine learning (ML) in medicine has transformed the ability to diagnose and predict conditions such as appendicular perforation. This work investigated how to predict perforation using clinical, biochemical, and ultrasound data using a variety of ML models, including logistic regression, random forest, and XGBoost. Non-linear correlations that conventional statistical approaches can miss might be found in complicated datasets processed by ML algorithms. XGBoost and random forest, which are renowned for their capacity to handle high-dimensional data and variable interactions, were used in this investigation.

## Materials and methods

Study design and patient selection

We retrospectively studied patients admitted to Safdarjung Hospital, New Delhi, India, between April 1, 2023, and April 31, 2024, reviewing the electronic medical records of patients with appendicular perforation. This study was conducted after the approval of the Institutional Ethics Committee (ref no. IEC/VMMC/SJH/Cert/jun-2024/65). The inclusion criteria were as follows: confirmed cases of appendicular perforation (postoperative status) and patients aged >18 years. The exclusion criteria included outside-operated patients and those with missing data. This analysis included 100 patients with a provisional diagnosis of appendicular perforation.

Data collection and definition of the variables

The collected data included age, gender, duration from symptom onset to admission, symptoms and signs (pain abdomen, fever, vomiting, abdominal guarding), blood test results (TLC, alkaline phosphatase (ALP), and serum bilirubin), and imaging results (maximum diameter of the appendix, presence of appendiceal faecolith, and presence of free fluid/peri-appendiceal collection).

Treatment and outcomes

We collected data on the duration of inpatient intravenous therapy and surgical procedures performed. Empirical antibiotic therapy included third-generation cephalosporins, piperacillin-tazobactam, and metronidazole. The need for surgical treatment was evaluated based on clinical expertise, an assessment of biochemical parameters, and hospital guidelines. A perforated appendix was characterized by a visible rent in the appendix or an appendicolith free within the abdominal cavity and the presence of purulent fluid (gross contamination) within the peritoneal cavity. Mortality included deaths that occurred in-hospital within 30 days of initial hospitalization.

Statistical analyses

All data were analyzed using the IBM SPSS Statistics for Windows, Version 28 (Released 2021; IBM Corp., Armonk, New York). Descriptive analysis was used to compare the differences in demographics, clinical manifestations, laboratory factors, and procedures performed. Continuous variables were presented as means (standard deviations (SDs)), while categorical data were presented as numbers (n) and percentages (%). Independent two-sample t-tests were used to analyze continuous variables. Categorical variables were compared using chi-square or Fisher's exact tests. The relationships among demographic characteristics, clinical manifestations, laboratory factors, and outcomes were assessed using univariate analysis. Multivariate logistic regression models in the forward selection mode were applied to significant factors from the univariate analysis. All statistical tests were two-sided; a p<0.05 was considered significant. ML models are used for non-linear variables and complicated datasets.

## Results

The primary goal of this analysis was to evaluate clinical, biochemical, and radiological predictors of appendicular perforation. Age (p=0.021), pulse rate (p=0.033), peri-appendiceal collection (p=0.030), and abdominal rigidity (p=0.050) were significantly associated with appendicular perforation. Peri-appendiceal collection emerged as a critical predictor, with an odds ratio (OR) of 7.09. Logistic regression and random forest provided modest predictive power, with area under the curve (AUC) scores of approximately 0.54-0.58 (Table 1).

**Table 1:** Key predictors and their inference with appendicular perforation TLC: total leukocyte count; ALP: alkaline phosphatase

Predictor	Coefficient	p-value	Interpretation
Age	0.6968	0.021	Significant predictor; older patients are more likely to have perforation.
Pulse rate	0.57	0.033	Higher pulse rates are associated with an increased risk of perforation.
Rigidity	0.4964	0.050	significant; patients with rigidity are more likely to experience perforation.
TLC	0.4754	0.074	Marginal association between higher TLC and perforation.
ALP	0.5955	0.057	Marginally significant; elevated ALP might indicate perforation.
Peri-appendiceal collection	0.5601	0.030	Presence of a peri-appendiceal collection shows a trend toward increased risk.

The demographic profile of patients with appendicular perforation reveals important trends that could influence treatment outcomes. The dataset highlights a mean age of 31 years, spanning from adolescence to seniors (13 to 69 years). Male patients slightly predominate, consistent with known appendicitis trends, with a male-to-female ratio of approximately 1.3:1. Pain onset varies, typically presenting 1-30 days before treatment, influencing the severity of cases. Fever, noted in many patients, aligns with an increased TLC and is indicative of infection or inflammation severity.

Demographic profile

**Figure 1:**
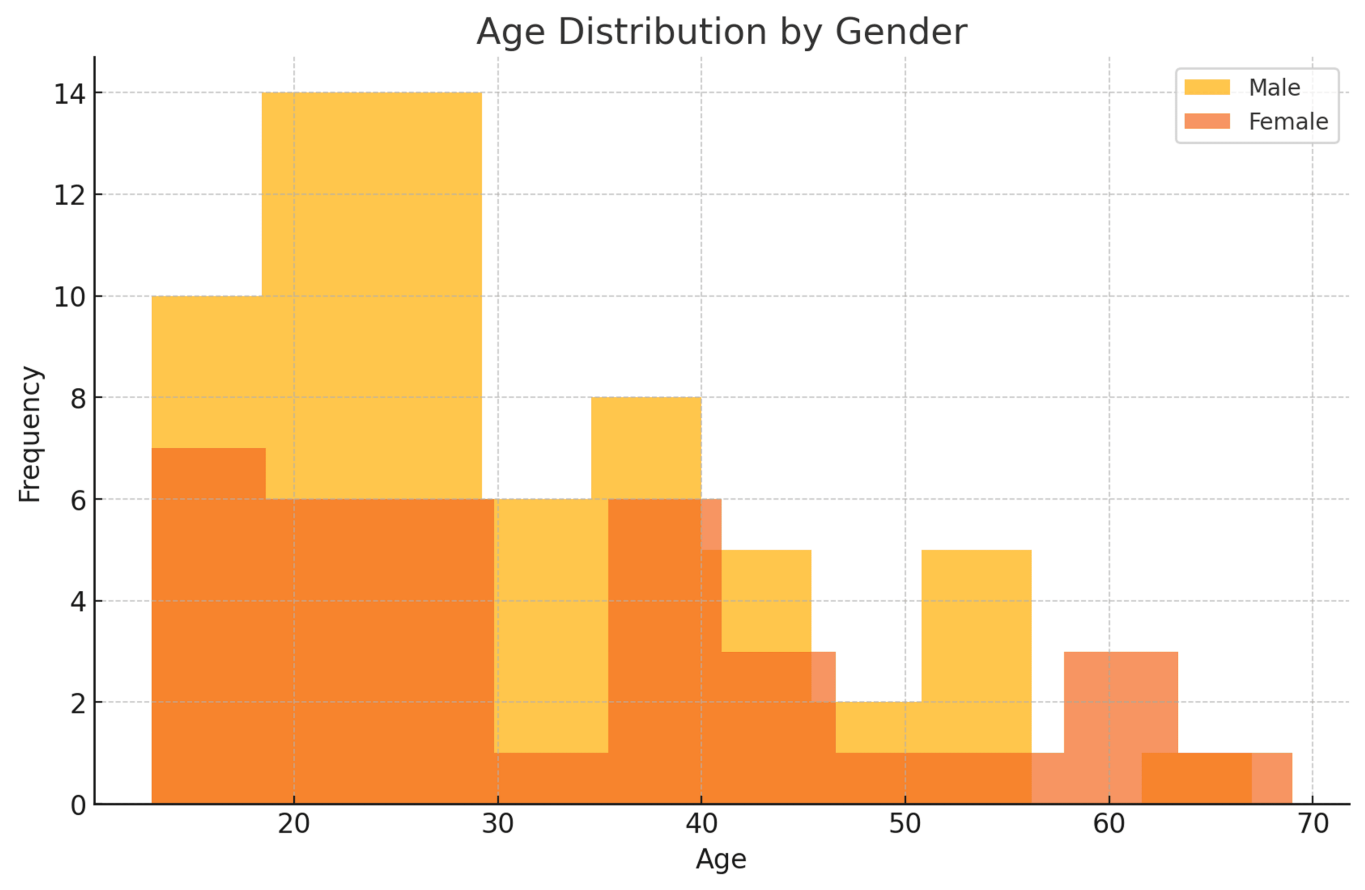
Age distribution by gender in cases of appendicular perforation Age in years

**Figure 2:**
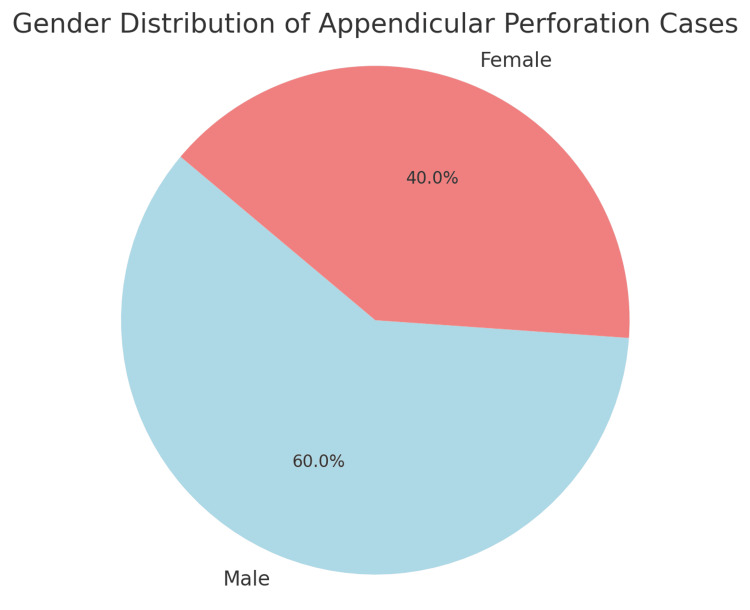
Gender distribution in cases of appendicular perforation

The highest incidence was in the 51-60 age group (N=6, 85% of cases), followed by 11-20 (N=10, 55%) and 31-40 (N=9, 47%). The 21-30 age group had fewer cases, but all were associated with perforation, although the absolute maximum number was present in the 21-30 age group (N=18) (Figure 1, Table 2). Male participants constituted 60% of the cases (N=30), while female patients represented the remaining 40% (N=20) (Figure 2, Table 3). Co-morbidity was present in 45 out of 50 perforation cases, suggesting a strong association. Most cases (52%) reported symptoms lasting three to five days. However, no linear relationship was found between the onset of pain and duration (Table 4).

**Table 2:** Age distribution

Age Group (Years)	Frequency	Percentage of Cases With Perforation	Cases With Perforation
11-20	18	55%	10
21-30	39	46%	18
31-40	19	47%	9
41-50	12	16%	2
51-60	7	85%	6
61-70	5	100%	5
71+	0	0%	0

**Table 3:** Gender distribution

Gender	Gender Cases	Gender Percentage
Male	30	60
Female	20	40

**Table 4:** Onset of symptoms and its distribution

Symptom Onset (Days)	Symptom Frequency	Symptom Percentage	Cases With Perforation
0-2	15	15	8
3-5	52	52	28
6-10	21	21	6
11-15	4	4	4
16-30	6	6	3

**Figure 3:**
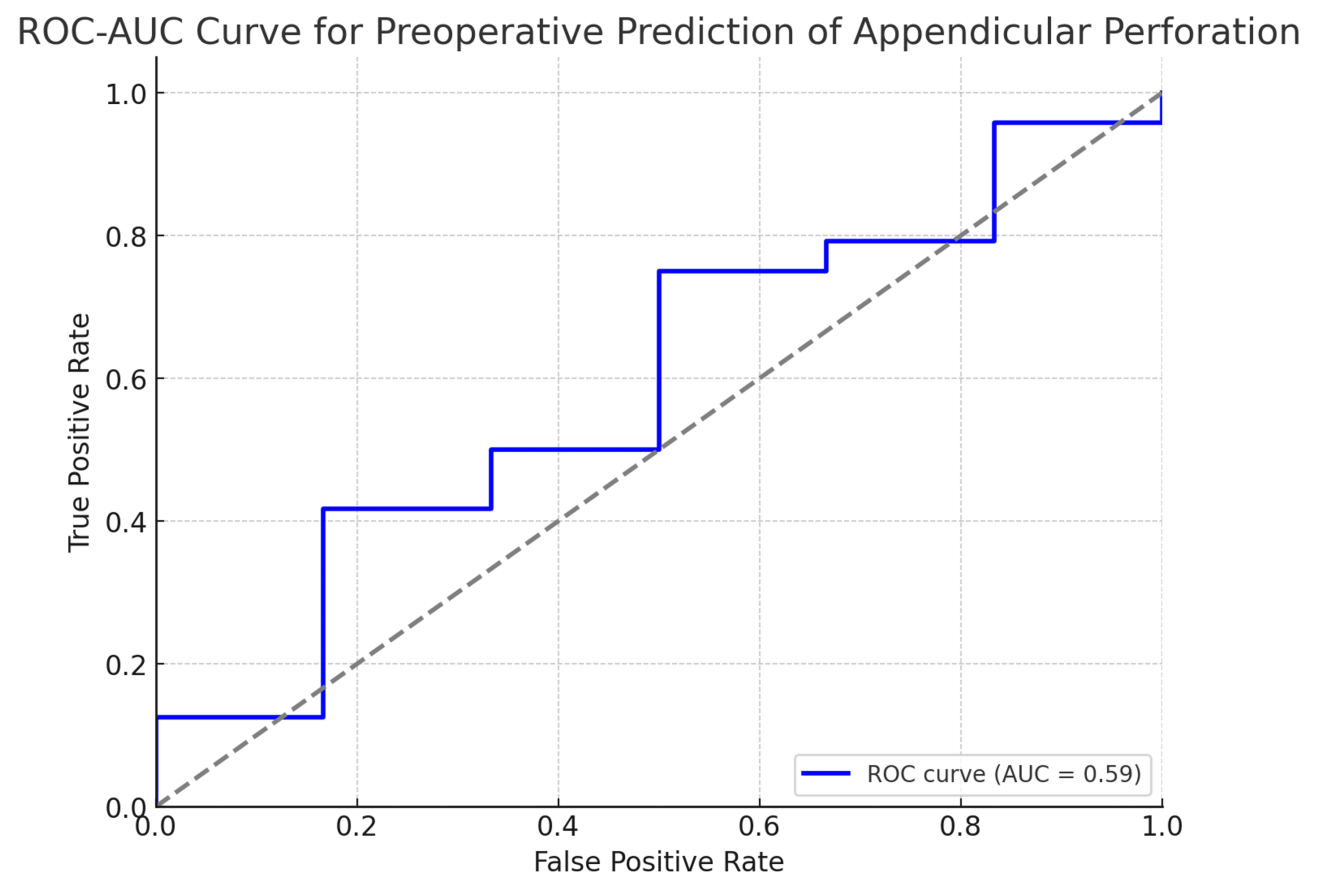
ROC-AUC curve for the preoperative prediction of appendicular perforation ROC-AUC: receiver operating characteristic/area under the curve

Here is the ROC-AUC curve for the preoperative prediction of appendicular perforation. The AUC value quantifies how well the model distinguishes between perforation and non-perforation cases: an AUC closer to 1 indicates excellent model performance, and an AUC around 0.5 implies a random guessing level. This plot allows us to visualize the trade-off between the true positive rate (sensitivity) and the false positive rate (Figure 3).

The AUC drop analysis highlights the predictors that have the greatest impact on the model's performance. An AUC score of 0.58 suggests moderate predictive power.

**Figure 4:**
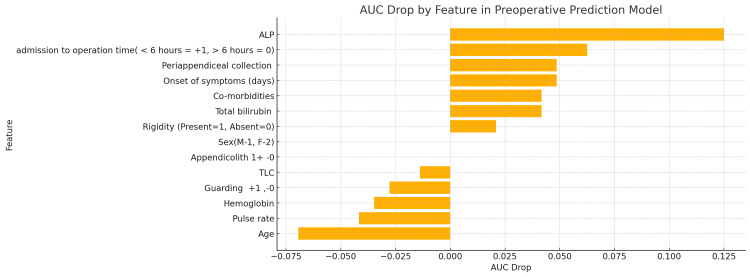
AUC drop curve by feature in the preoperative prediction model This chart shows how much the AUC decreases when each feature is removed. The higher the AUC drop, the more critical that feature is for predicting appendicular perforation. AUC: area under the curve

In this study, we investigated the utility of various clinical, biochemical, and radiological parameters in predicting appendicular perforation. The logistic regression analysis identified peri-appendiceal collection as a notable predictor, with a significant OR suggesting a higher likelihood of perforation when this ultrasound finding is present. Other clinical markers, such as pulse rate and TLC, also showed potential utility, indicating an increased risk, albeit not reaching statistical significance. The ML models, including random forest and XGBoost, were applied to validate these findings, each demonstrating moderate predictive power with AUC scores around 0.54 to 0.58 (Figure 4).

## Discussion

Age and gender profile

The current analysis identified that appendicular perforation was most common in the 21-30 age group, with a notable incidence in the 31-40 age group as well. This aligns with studies such as Bhangu et al. [10], which found that younger adults are at higher risk due to delayed presentations and more aggressive disease progression. Additionally, the male predominance seen in this study (60% of cases) is consistent with findings reported by Lee et al. [11], which noted a higher incidence of complicated appendicitis, including perforation, in males.

Symptoms and signs

The distribution of onset of symptoms showed that most perforation cases presented after three to five days of symptoms. Previous studies, including those by Kim et al. [12], support that delayed presentation beyond 48 hours is a strong risk factor for appendicular perforation. This reinforces the importance of timely diagnosis and surgical intervention. Guarding was observed in a significant proportion of perforation cases (28 cases), aligning with studies that identify guarding as a hallmark of advanced appendicitis. However, rigidity was less frequently noted, suggesting that while it may indicate severe cases, it is not as common as guarding. This finding is comparable to data reported by Echevarria et al. [13], which also highlighted variability in the presentation of physical signs.

Laboratory findings

The analysis showed that higher TLC levels were associated with perforation (p = 0.074), which is consistent with studies by Andersson et al. [14], noting that leukocytosis is a reliable marker of inflammation and advanced appendicitis. On the other hand, ALP levels did not show a significant association (p = 0.057), suggesting limited diagnostic utility, as corroborated by prior research indicating that ALP, although raised in some cases, is not a primary marker for appendicitis complications.

Ultrasound findings

Peri-appendiceal collection was significantly associated with a higher risk of perforation (p = 0.030). This is supported by Becker et al. [15], who emphasized that the presence of fluid collections on imaging should raise suspicion for perforation. The presence of an appendicolith did not significantly correlate with perforation, which aligns with mixed results in the literature, where appendicolith is seen as a risk factor but not a definitive predictor.

The analysis underscores the challenges of predicting appendicular perforation preoperatively using available clinical and ultrasound data, highlighting the need for integrating more comprehensive diagnostic indicators and advanced modeling techniques to enhance prediction accuracy.

Logistic regression and random forest provided modest predictive power, with AUC scores around 0.54-0.58. The models suggest that the available preoperative data, while useful, may not fully capture the complexity of predicting perforation.

Strengths and limitations

The merits of this study lie in its provision of insights into the utility of ultrasound findings, such as peri-appendiceal collection, and its exploration of ML methods to enhance preoperative predictions. Multiple models were evaluated to ensure robust comparisons.

The limitations of this study indicate that preoperative prediction using the available data remains challenging, as reflected in the modest AUC values. Other clinical variables, such as inflammatory markers (e.g., CRP), or imaging modalities, might improve prediction. However, computational issues limited the deeper exploration of complex models like XGBoost.

## Conclusions

Early identification of patients at risk for appendicular perforation is crucial for improving clinical outcomes and minimizing complications associated with acute appendicitis. The review highlights several key clinical, biochemical, and radiological factors that can aid in predicting perforation risk. Clinical indicators such as prolonged symptom duration, fever, and signs of peritonitis, combined with elevated biochemical markers like WBC count, offer valuable insights into the likelihood of perforation. Furthermore, radiological imaging, particularly ultrasound, plays a pivotal role in confirming the diagnosis and detecting complications. Understanding these predictive factors can guide timely surgical intervention, ultimately reducing morbidity and mortality rates associated with appendiceal perforation. Future research should focus on refining these diagnostic tools and exploring additional biomarkers to enhance early detection and improve patient outcomes.

## References

[1] McGregor J, Thompson G (2016). Are guidelines being adhered to in the radiological investigation of appendicitis in our paediatric and adult population?. Clin Radiol.

[2] Bonadio W, Shahid S, Vardi L, Buckingham C, Kornblatt A, Free C, Homel P (2018). A pre-operative clinical scoring system to distinguish perforation risk with pediatric appendicitis. J Pediatr Surg.

[3] Vaswani Vaswani, KK KK (2002). The normal appendix and appendicitis and its complications. Contemp Diagn Radiol.

[4] Wilkie DP (1914). Acute appendicitis and acute appendicular obstruction. Br Med J.

[5] Shogilev DJ, Duus N, Odom SR, Shapiro NI (2014). Diagnosing appendicitis: evidence-based review of the diagnostic approach in 2014. West J Emerg Med.

[6] Amer E (2021). Mimickers of acute appendicitis. Doubts, Problems and Certainties About Acute Appendicitis.

[7] Lo HC, Chien WK (2016). Does age affect the outcomes and management of pediatric appendicitis in Taiwan?. Formos J Surg.

[8] Kim M, Kim SJ, Cho HJ (2016). Effect of surgical timing and outcomes for appendicitis severity. Ann Surg Treat Res.

[9] Onur MR, Akpinar E, Karaosmanoglu AD, Isayev C, Karcaaltincaba M (2017). Diverticulitis: a comprehensive review with usual and unusual complications. Insights Imaging.

[10] Bhangu A, Søreide K, Di Saverio S (2015). Acute appendicitis: modern understanding of pathogenesis, diagnosis, and management. Lancet.

[11] Lee SL, Ho HS (2006). Acute appendicitis: is there a difference between children and adults?. Am J Surg.

[12] Kim HY, Park JH, Lee YJ (2018). Risk factors for perforation in patients with suspected acute appendicitis. Annals of Surgery.

[13] Echevarria S, Rauf F, Hussain N (2023). Typical and atypical presentations of appendicitis and their implications for diagnosis and treatment: a literature review. Cureus.

[14] Andersson RE, Hugander A, Ravn H (2019). Diagnostic accuracy and laboratory findings in acute appendicitis. World J Surg.

[15] Becker T, Kharbanda A, Bachur R (2020). Imaging strategies in appendicitis and the role of appendicolith in predicting complications. Pediatric Emergency Care.

